# A Case of Shoulder Presentation, with Prolapse of Cord

**Published:** 1861

**Authors:** H. Webster Jones


### A CASE OF SHOULDER PRESENTATION, WITH PROLAPSE OF CORD. VERSION BY EXTERNAL MANIPULATION. TREATMENT BY POSITION. DEATH OF CHILD.

By H. WEBSTER JONES, A. M., M. D.

I was called at 7 P. M., January 28th, 1861, to see Mrs. A. P., primipara æt 16.

The patient was upon the bed, suffering with severe labor pain, her clothing, the bed, and even the floor saturated with the liquor amnii, which had just been discharged in great quantity and with considerable force.

An examination detected the left scapula presenting, and a lengthened *prolapsus funis* protruding under the axilla through a dilatable os.

Manipulation of the child, through the abdominal and uterine walls, directing extremity (upward and inward), the (cephalic downward and inward,) resulted, after some time, in a complete correction of the mal-position, the head now engaging with the vertex towards the left acetabulum.

The cord, still largely prolapsed, and pulsating strongly, was now carried toward the right acetabulum, where it was least liable to pressure, and the patient assumed at my request the position upon the knees and chest, recommended by Dr. T. Gaillard Thomas.

During two severe pains, I attempted to maintain the fallen loop of cord within the uterus and beyond the head, but without success, when the patient refused utterly to bear another in that position.

The head at 3 A. M. bore upon the perineum, and checked materially the force of the fœtal circulation. Spasmodic movements of the child soon occurred, indicating its danger.

Another trial of Thomas' position failed, the pains pressing down in one direction when well supported in another, and at 4.30 A. M. the child was delivered, there having been no perceptible pulsation in the cord for nearly an hour. The usual means of resuscitation were resorted to, but proved unavailing.

The noticeable features of this case, are

1st, the probable effect of a sudden discharge of liquor amnii when in excess, as determining the mal-positions, as well as the *prolapsus funis*.

2d, the safe and efficient manner in which version was accomplished, under the conjoined and unfavorable circumstances of evacuated waters and a strongly contracting uterus;

3d, the discomfort and distress produced by the prone position, rendering it *in this case*, unavailable toward the restoration of the funis to the uterine cavity.

